# Elevated Serum MCP‐2 and TARC Associated With Increased Risk of Death in Guamanian ALS Patients

**DOI:** 10.1111/ene.70088

**Published:** 2025-02-25

**Authors:** Risana N. Chowdhury, Mus'ab A. Azam, Suhaib A. Azam, Shteynman Lana, Erin N. Culver, Ralph M. Garruto, Katherine Wander

**Affiliations:** ^1^ Department of Anthropology Binghamton University (SUNY) Binghamton New York USA; ^2^ State University of New York Upstate Medical University Syracuse New York USA; ^3^ University at Buffalo School of Medicine and Biomedical Sciences Buffalo New York USA; ^4^ Renaissance School of Medicine at Stony Brook University Stony Brook New York USA; ^5^ Colorado Center for Personalized Medicine University of Colorado‐Anschutz Medical Campus Aurora Colorado USA; ^6^ Department of Biological Sciences Binghamton University (SUNY) Binghamton New York USA

**Keywords:** Guam ALS‐PD, lytico‐bodig, neurodegeneration, neurodegenerative disease, serum inflammation

## Abstract

**Background:**

This study explores the relationship between inflammation and longevity in a high‐incidence focus of amyotrophic lateral sclerosis (ALS) in post‐WWII Guam. Characteristics of this focus include the sudden appearance of the disease in high numbers and the unusually long lifespan (without medical interventions) seen in some cases. We used bio‐banked specimens to evaluate the relationship between serum immunoregulators and survival time.

**Methods:**

We evaluated sera from 69 Guam ALS cases collected within 2 years of symptom onset by NIH researchers from 1950 to 1983 for 11 immunoregulators via ELISA (CRP, eotaxin‐1, RANTES, IL‐6, IL‐8, IL‐10, IFN‐γ, IP‐10, MCP‐1, MCP‐2 and TARC). Factor analysis identified two factors responsible for ~68% of the variation in the data. We estimated Cox proportional hazards models to identify immunoregulators associated with time to death.

**Results:**

Each 10‐unit increase in factor 2 cytokines (MCP‐2 and TARC) was associated with a 38% increase in the risk of death (HR: 1.38; 95% CI: 1.19, 1.65; *p*: 0.00). *Discussion*: Like sporadic ALS cases worldwide, inflammation is associated with a shortened lifespan in Guamanian ALS; more specifically, our findings suggest serum levels of MCP‐2 and TARC at onset may predict disease duration. Further investigation is needed to determine the role of these immunoregulators in disease prognosis and as targets for diagnostic and therapeutic interventions.

## 1 Introduction

This research explores the role of inflammation in a mysterious focus of amyotrophic lateral sclerosis (ALS) among Indigenous CHamoru living in post‐WWII Guam. Most research on Guamanian ALS has focused on disease causality; less attention has gone to an intriguing aspect of the disease: the length of survival post‐onset of initial symptoms. While most ALS patients in the US live 2–4 years after diagnosis, some Guamanian patients lived in excess of 20 years after diagnosis, with no modern medical treatment [1, 2]. In ALS cases occurring in industrialized parts of the world, high inflammation is associated with a shortened lifespan. This research investigates the relationship between inflammation and survival time in ALS cases in the high infectious disease environment of mid‐twentieth‐century Guam.

**FIGURE 1:**
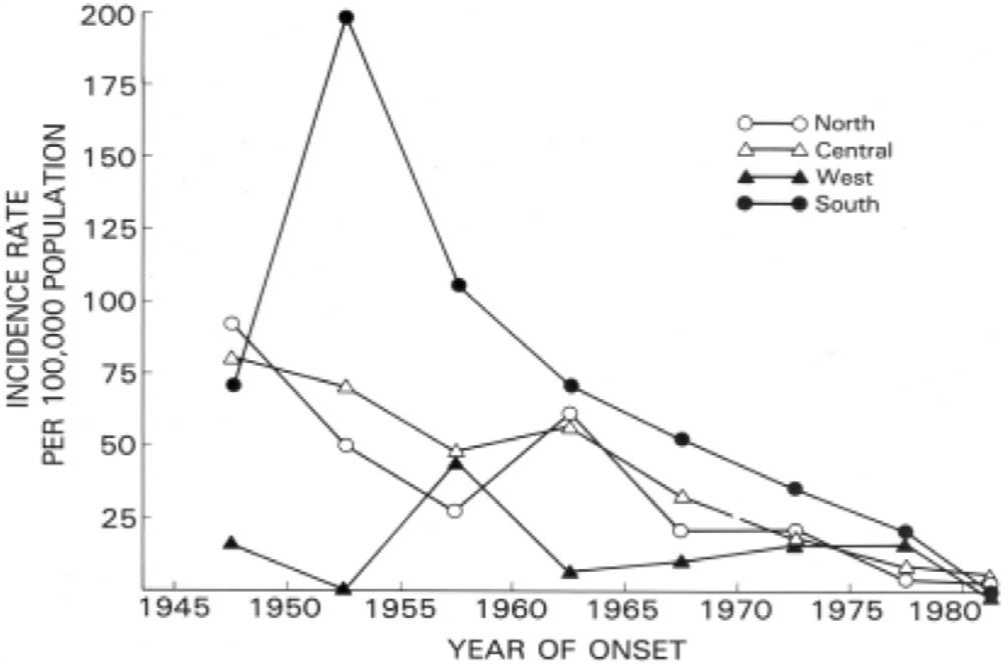
Decline of lytico‐bodig on Guam by location [6].

**FIGURE 2:**
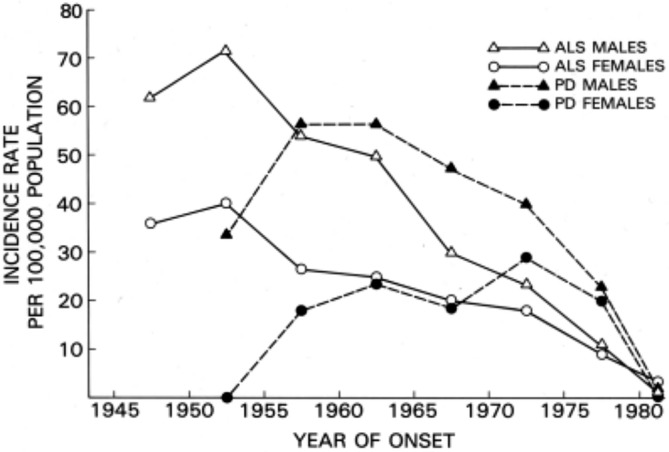
Decline of lytico‐bodig on Guam by sex [6].

Following the Japanese occupation of Guam during World War II, two sets of neurological ailments constituted leading causes of death among the CHamoru (nearly all Indigenous Guamanians belong to this ethnic group) [3, 4]. Patients developed symptoms—a combination of tremors and dementia or progressive loss of motor function—at a rate of 80–100 times that of the worldwide average. Guamanians referred to these phenomena as *lytico‐bodig*. They were later identified as ALS and Parkinsonism dementia (PD) [5]. With modernization on Guam, the incidence of both diseases diminished over time (Figures 1 and 2). The last remaining cases numbered around 10–25 individuals between 1980 and 1991 [6, 7].

**FIGURE 3:**
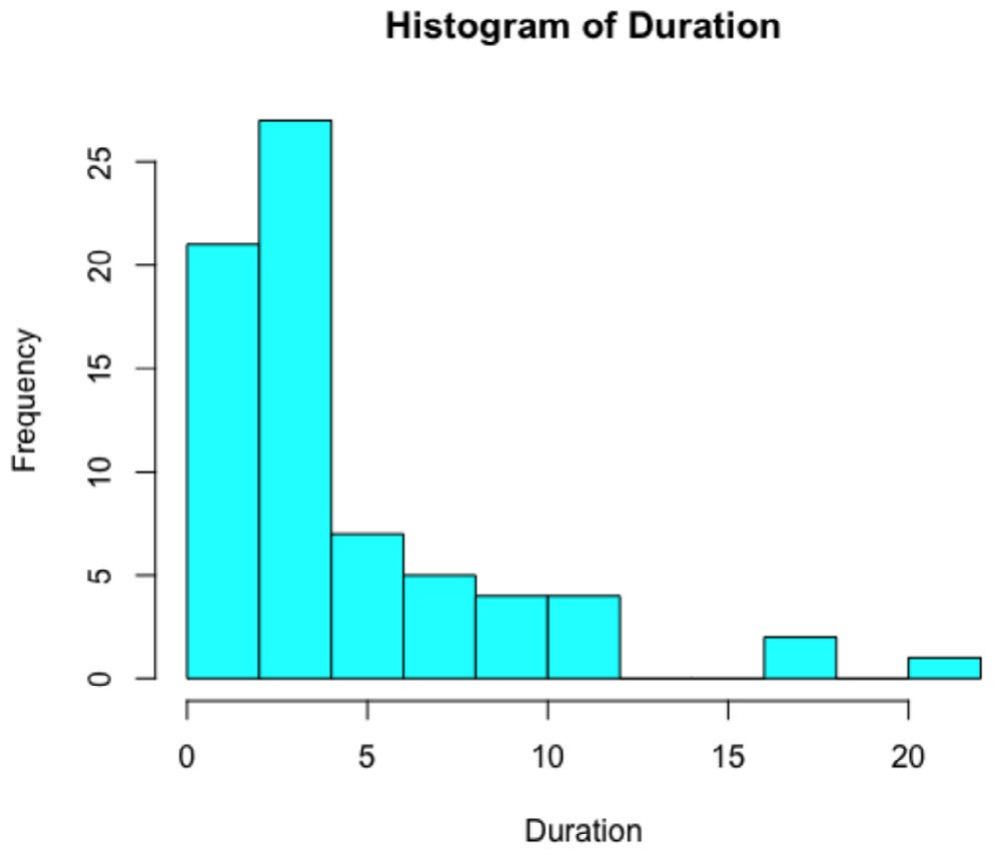
Cases represent a broad range of survival times (1–22 years, *n* = 69).

Amyotrophic lateral sclerosis is an upper and lower motor neuron disorder in which patients progressively lose muscle function. Symptoms often begin with slurred speech, progressing to total loss of motor function. Without respiratory aid, loss of lung function is the most common cause of demise in patients. There are two categories of ALS: sporadic and familial. In cases of familial ALS, patients inherit the disease, typically in an autosomal dominant manner [8]. The superoxide dismutase (SOD1) mutation associated with familial ALS was absent in Guamanian ALS patients [9]. In cases of sporadic ALS, in which patients have no family history of the disease, the typical survival time is 2–5 years, with only 10%–20% of patients surviving over 10 years (Figure 3). Guamanian ALS symptoms were nearly identical to those present in ALS cases worldwide [6, 7, 10].

### 1.1 Clinical and Neuropathological Features of ALS and PD on Guam

For the most part, Guamanian ALS was clinically indistinguishable from other forms of ALS. Clinical features of Guamanian ALS were progressive muscular atrophy, lateral sclerosis, bulbar paralysis, and pseudobulbar palsy, involving upper and lower motor neurons [1, 6]. The primary differences between Guamanian and sporadic ALS were survival time: the mean survival time after diagnosis was longer in Guamanian ALS (4.2 years) than in sporadic ALS (2.5 years) [7, 11]. Guamanian ALS patients sometimes lived an unusually long time (20+ years) without clinical intervention [6, 7]. Previous immunological studies of serum IgA, IgG, and HLA antigen levels among Guamanian ALS cases were conducted in the 1970′s, but immunoregulators and disease duration have never been analyzed [11, 12, 13, 14, 15]. Inflammation has been found to predict ALS patient lifespan in industrialized populations [16, 17]. We address whether inflammation predicts survival time in Guamanian ALS.

## 2 Methods

### 2.1 Study Design

We assessed inflammation in serum specimens collected on Guam from 1950 to 1983, when Guamanian ALS patient monitoring and follow‐up ended. Serum immunoregulators evaluated were: C‐reactive protein (CRP); interleukins (IL) ‐6, ‐8, and ‐10; interferon‐gamma (IFN‐γ) and interferon‐gamma inducible protein‐10 (IP‐10); monocyte chemoattractant proteins (MCP) ‐1 (CCL2) and ‐2 (CCL8); eotaxin‐1 (CCL11); regulated on activation, normal t‐cell expressed and secreted (RANTES, CCL5) and thymus and activation‐regulated chemokine (TARC, CCL17) (Table 1).

**TABLE 1:** Immunoregulator function in ALS.

Immunoregulator	Name	In ALS
MCP‐1	Monocyte chemoattractant protein 1; CCL2	Elevated in ALS patients [18]
MCP‐2	Monocyte chemoattractant protein 2; CCL8	Elevated in serum of ALS patients; found in glial cells containing (*m*)SOD1 in familial ALS cases [19, 20]
RANTES	Regulated on activation, normal T cell expressed and secreted; CCL5	Associated with faster disease progression/shorter ALS patient lifespan [15, 21]
TARC	Thymus and activation‐regulated chemokine; CCL17	Unknown
Eotaxin‐1	CCL11	Higher in ALS patients than in non‐inflammatory neurological disease patients [21, 22]
IFN‐γ	Interferon‐gamma	Elevated in ALS patient CSF and serum [15, 21, 23, 24]
IP‐10	Interferon‐gamma inducible protein‐10; CXCL10	May be associated with ALS longer disease duration [21]
IL6	Interleukin‐6	Elevated in ALS cases, especially later in disease; positive correlation with hypoxemia [21, 22]
IL‐8	Interleukin‐8; CXCL8	Increased in ALS patient serum and CSF [21]
IL‐10	interleukin‐10; human cytokine synthesis inhibitory factor	Increased in ALS patients/associated with longer disease duration [15, 21]
CRP	C‐reactive protein	Elevated in ALS patient serum later in disease [21]

At the time of collection, all specimens were obtained with informed written consent from patients as part of the NIH (NINDS) Research Center program on Guam (Protocol Number 83‐N‐34) following the ethical standards set forth by the 1947 Nuremberg Code and the 1965 Declaration of Helsinki. The analyses presented here are consistent with the original use for which the samples were collected.

### 2.2 Participants

Serum specimens from ALS cases used for this study were collected between 1962 and 1983 (age at onset 27–72 years; Table 2) from CHamoru males and females residing on Guam. The 11 serum immunoregulators analyzed for this study were previously validated for stability in archived frozen human sera [25, 26]. Cases with reported year of birth, year of onset, and year of death (or survival until 1983) were considered for inclusion. Only liquid sera kept consistently frozen (aside from short periods of thawing during aliquot retrieval) in intact vials were used. Specimens were excluded from analyses if records indicated desiccation or prolonged periods of thaw. We used archival records to exclude participants with malaria, diabetes, and symptomatic acute infectious diseases at the time of specimen collection. Specimens were selected from individuals whose serum was collected within 2 years of disease onset to offset the deleterious effects of later‐stage ALS on immune function [23, 24]. Sex was self‐reported at the time of blood draw; participants were given a choice between male and female, as was standard practice at the time.

**TABLE 2:** Baseline characteristics of Guamanian ALS patients (*n* = 69).

Sex	
Male	43 (62.3%)
Female	26 (37.7%)
Location of village	
North	14 (20.3%)
Central	31 (44.9%)
South	21 (30.4%)
Age of disease onset	
< 30 years	1 (14.5%)
30–50 years	38 (55.1%)
50–70 years	28 (40.6%)
> 70 years	2 (2.9%)

### 2.3 Laboratory Analyses

Immunoregulator levels were evaluated using a custom 10‐plex cytokine (Quansys Bioscience) and single‐plex CRP (Biocheck BC‐1119 HS CRP EIA) enzyme immunoassay. Intra‐assay coefficients of variation (CVs) were 5.3% for eotaxin‐1, 8.4% for IL‐6, 9.18% for IL‐8, 11.1% for IL‐10, 8.4% for IFN‐γ, 9.1% for IP‐10, 2.5% for MCP‐1, 4.3% for MCP‐2, 8.0% for TARC, and 4.9% for CRP. Over half of the specimens resulted in %CV for RANTES above 20% despite multiple reruns. Therefore, data for this immunoregulator was excluded.

### 2.4 Statistical Analysis

We analyzed data with R statistical software [27]. DFBETAS tests [28] were used to identify influential data points; these were excluded from analysis. To present the most readily interpretable effect estimates (e.g., the effect of a 10 pg/mL increase in cytokine concentration), we divided all immunoregulator concentrations by 10 prior to modeling. We used principal components analysis to identify factors; then we used factor analysis to identify immunoregulators loading above 50% on each factor and constructed factor variables by summing the centered values for each immunoregulator in that factor [29].

We estimated crude and adjusted Cox proportional hazards (CPH) models [30] to assess the effects of inflammation in the early years after diagnosis on the hazard of death.

### 2.5 Role of the Funding Source

The funder of the study had no role in study design, data collection, data analysis, data interpretation, or writing of the report.

## 3 Results

Sixty‐nine ALS cases (43 males and 26 females) met the inclusion criteria; four participants were still alive in 1983. Mean age of onset was 49.8 years for females (median age, 47 years; range 30–71) ALS patients and 48 years for males (median age, 48 years; range 33–72) ALS patients in this study. Duration (Figure 3) averaged 5.1 years (ranging from 1 to 18 years) for female cases and 4.4 years (ranging from 1 to 22 years) for male cases. The mean age of onset for patients who lived at least 10 years after disease onset was 38.3 years (median age, 37 years; range, 27–50). Total mean survival time in this sample was 4.75 years (range of survival was 1–22 years). Older age of disease onset was associated with a higher risk of death; sex and location of residence on the island were unassociated with the risk of death (Table 3).

**TABLE 3:** Crude models of demographic variables and death among Guamanian ALS cases.

	HR	95% CI	Increase in risk of death	*p*
Age at onset	1.03	1.00, 1.05	3%	**0.02**
Sex	1.18	0.72, 2.00	18%	0.48
Location	1.35	0.95, 1.92	35%	0.97

*Note:* Age at onset was significantly associated with survival time, while sex and location (north, central, south Guam) were not. Bolded *p*‐values ≤ 0.05 represent statistical significance.

**FIGURE 4:**
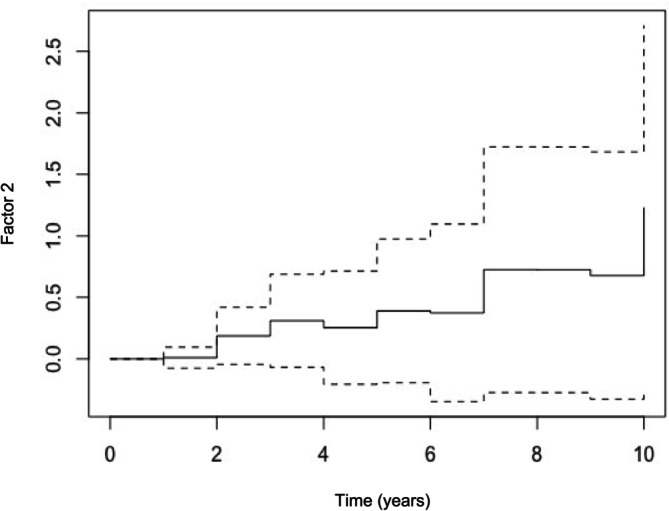
Kaplan–Meier Survival curve depicts the change in the probability of death over time for Factor 2 (MCP‐2 and TARC). Horizontal lines depict the duration of survival in years; vertical lines depict the change in the probability of death; intermittent lines represent 95% confidence intervals. *n* = 61 events, 69 cases total.

Explaining 68% of the variation in immunoregulators, Factor 1 included IL‐6, IL‐8, and IL‐10; Factor 2 included MCP‐2 and TARC. Crude CPH models suggested associations between death and IFN‐γ, IP‐10, IL‐6, MCP‐1, MCP‐2, TARC (Table 4), and Factor 2, but not Factor 1 (Table 5). After controlling for age, the association between Factor 2 and death persisted, such that a 10‐unit increase in Factor 2 was associated with a 39% increase in hazard for death (HR: 1.39, 95% CI: 1.18, 1.64, *p*: 0.00; Table 5 and Figure 4). After controlling for age, independent associations between MCP‐2 (HR: 1.32, 95% CI: 1.11, 1.56, *p*: 0.00) and TARC (HR: 1.02, 95% CI: 0.99, 1.03, LR *p*: 0.09) and death were also apparent, as was an association between IP‐10 and death (with additional control for IFN‐γ; HR: 1.20, 95% CI: 1.06, 1.37, *p*: 0. 00) (Table 5).

**TABLE 4:** Crude models of immunoregulators and death among Guamanian ALS cases.

Immunoregulator	HR	95% CI	Increase in risk of death	*p*	Events
CRP	1.01	0.92, 1.10	0.5%	0.91	61
IFN‐γ	2.34	1.25, 4.36	134%	**0.01**	61
IP‐10	1.18	1.07, 1.31	18%	**0.00**	63
IL‐6	1.05	0.10, 1.11	5%	**0.06**	63
IL‐8	1.00	0.99, 1.00	0%	0.94	63
IL‐10	1.03	0.80, 1.33	2.9%	0.81	61
MCP‐1	1.03	1.00, 1.06	3%	**0.02**	61
MCP‐2	1.24	1.08, 1.44	24%	**0.00**	61
TARC	1.03	1.01, 1.04	2.8%	**0.00**	61
Eotaxin‐1	1.00	0.99, 1.02	0.3%	0.71	64
Factor 1	1.07	0.90, 1.09		0.90	
Factor 2	1.38	1.19, 1.65	38%	**0.00**	

*Note:* Serum levels of IFN‐γ, IP‐10, MCP‐1, MCP‐2, IL‐6 and TARC were associated with increased risk of death. “Events” refers to number of events for each immunoregulator after removal of influential data points and censored cases. Bolded *p*‐values ≤ 0.05 represent statistical significance.

**TABLE 5:** Crude and adjusted models of immunoregulators and death among Guamanian ALS cases.

Model	Hazard ratio	95% CI	Increase in risk of death	*p*	Likelihood ratio
Factor 2	1.38	[1.19, 1.65]	38%	**0.00**	—
Factor 2	1.39	[1.18, 1.64]	39%	**0.00**	**LR: 0.002**
Age	0.99	[0.97, 1.03]	−1%	0.81	
MCP‐2	1.32	[1.11, 1.56]	32%	**0.00**	**LR: 0.003**
TARC	1.02	[0.99, 1.03]	1.4%	0.09	
Age	0.99	[0.96, 1.03]	−1%	0.66	
IFN‐γ	1.02	[0.43, 2.39]	2%	0.97	**LR: 0.006**
IP10	1.2	[1.06, 1.37]	20%	**0.00**	
Age	1.03	[1.00, 1.06]	3%	**0.03**	
IL‐6	1	[0.86, 1.17]	0%	1.00	LR: 0.4
IL‐8	0.996	[0.99, 1.01]	−0.4%	0.47	
IL‐10	1.26	[0.83, 1.92]	26%	0.28	
Age	1.02	[0.99, 1.05]	2%	0.12	

*Note:* Adjusted for age, a 10‐unit increase in Factor 2 was associated with a 39% increased hazard for death. Increased serum IFN‐γ, MCP‐2 and IP‐10 were associated with highest increase in risk of death per year. Time to death was unassociated with Factor 1 (IL‐6. IL‐8 and IL‐10) (HR: 1, 95% CI [0.9, 1.1] LR *p*: 0.3); eotaxin‐1 (HR: 1, 95% CI [0.99, 1.02], LR *p*: 0.7), CRP (HR:1, 95% CI [0.92, 1.1], LR *p*: 0.9), IL‐8 (HR:1, 95% CI [0.99, 1], LR *p*: 0.9) and IL‐10 (HR: 1.03, 95% CI [0.8, 1.33], LR *p*: 0.8). Bolded *p*‐values ≤ 0.05 represent statistical significance.

## 4 Discussion

We found a strong positive association between inflammation and decline to death among Guamanian ALS patients, such that those with higher inflammation early in the course of the disease died sooner. Our analyses focused on a subset of immunoregulators that we found to be robust to long‐term archival [25, 26] rather than the full set of immunoregulators involved in inflammatory pathways. Because of this, we cannot fully distinguish between a general effect of inflammation and a particular pathway. However, our analyses do point to two immunoregulators in particular: together, the pro‐inflammatory immunoregulators MCP‐2 and TARC were particularly strongly associated with death among Guamanian ALS patients (Figure 4). This largely accords with findings from industrialized settings that inflammation is positively associated with—and may contribute to—disease progression. Little is known about the specific roles MCP‐2 and TARC may play in ALS disease etiology and progression. The only reported association between MCP‐2 and ALS is the overexpression of MCP‐2 (along with CXCL17 and RANTES) in mutated (*m*)SOD‐1 glial cells [19]. (*M*)SOD‐1 is a characteristic of familial ALS, although there is some debate about its presence and role in sporadic ALS [20, 31]. To date, no (*m*)SOD‐1 has been found in Guam ALS [4]. However, in the context of the role of (*m*)SOD‐1 and MCP‐2 in familial ALS, our findings suggest that assessing serum levels of MCP‐2 and/or TARC in the early stages of the disease may be informative for sporadic ALS disease prognoses.

In sporadic ALS, IL‐6 and IL‐10 are part of the inflammatory cascade and are elevated in comparison to non‐ALS controls. However, like other studies [18, 22], we found that neither was a predictor of disease progression or survival. Serum IL‐6 levels increase with increased hypoxemia (low blood oxygen levels). Increased IL‐6 is also positively associated with hypermetabolism [21] and decreased force vital capacity (%FCV) [21] in sporadic ALS. Serum IL‐6 levels increase in the later stages of the disease in sporadic ALS [15]. Our results also support these reported findings, as IL‐6 and IL‐10 are not elevated in our sample.

Interferon‐γ has been found to be positively associated with spinal cord neurodegeneration and disease progression in sporadic ALS [32] Consistent with these findings, we observed a positive association between a related protein, IP‐10 (controlled for age and IFN‐γ), and the risk of death. The IFN‐γ pathway may be another important prognostic tool and target for therapeutic intervention in treating ALS.

While some studies have found positive associations between CRP and ALS disease progression (e.g., greater functional impairment, shorter survival response to immune‐regulating treatments) [17], we did not find an association between CRP and ALS progression. Our sample consisted of specimens collected early in the course of the disease, whereas CRP may play a larger role in disease progression later in the course of the disease. It will be important to continue to consider multiple points in proinflammatory pathways as research continues to investigate the role of inflammation in ALS disease progression.

Younger age at onset is a predictor of longer survival in sporadic ALS cases [18]. For example, patients who develop sporadic ALS before age 40 often live longer than 10 years post onset [18]. We found a similar pattern of increasing risk for death with older age at disease onset. Early age at onset may partly explain the unusually long mean survival time characteristic of Guamanian ALS cases [5].

Psychosocial factors may play a critical role in patient survival after the onset of disease. In sporadic ALS, decreased self‐esteem and increased stress, depression, hopelessness, and low social support seem to shorten survival time, and longer survival is seen in married sporadic ALS patients compared to those who live alone [18, 33]. In Guam, the strong social support and care dedicated to Guamanian ALS patients by friends and family were noted by researchers over the decades. ALS patients often lived at home and were visited regularly by friends and family [34].

### 4.1 Limitations

We selected immunoregulators based on stability over long‐term freezer storage [25], which may have led to the exclusion of important and informative immunoregulators from the analysis; thus, we can point to inflammatory pathways in general, but not direct roles for these particular immunoregulators. We were only able to access adequate quality specimens from 69 participants; this limited sample size may lead to imprecision. We relied on archived specimens, which were not collected with these specific analyses in mind; this could introduce some imprecision or bias.

### 4.2 Implications for Future Research and Clinical Care

Our findings shed light on the critical role of inflammation in ALS progression and longevity. The identification of MCP‐2 and TARC as potential immunoregulators for predicting survival and disease progression in ALS patients may lead to advancements in understanding the pathophysiology of the disease. These immunoregulators, and inflammatory pathways more generally, hold promise for clinical applications, offering the possibility of more personalized prognostic assessments and treatment strategies in newly diagnosed ALS patients. Understanding neurodegenerative pathways associated with MCP‐2, TARC, and inflammation overall could also lead to the development of preventive interventions for at‐risk individuals with a family history of ALS.

## Author Contributions

**Risana N. Chowdhury:** conceptualization, investigation, funding acquisition, writing – original draft, methodology, visualization, writing – review and editing, software, formal analysis, project administration, data curation, supervision, resources. **Mus'ab A. Azam:** investigation, writing – original draft, writing – review and editing. **Suhaib A. Azam:** investigation, writing – review and editing, writing – original draft. **Shteynman Lana:** project administration, investigation, data curation. **Erin N. Culver:** investigation, writing – review and editing, project administration, supervision. **Ralph M. Garruto:** conceptualization, supervision, resources. **Katherine Wander:** conceptualization, writing – review and editing, methodology, software, supervision, resources.

## Conflicts of Interest

The authors declare no conflicts of interest.

## Data Availability

The dataset (under embargo until January 31, 2026) will be shared with researchers for secondary analysis upon reasonable request.
